# Catgut embedding in acupoints combined with repetitive transcranial magnetic stimulation for the treatment of postmenopausal osteoporosis: study protocol for a randomized clinical trial

**DOI:** 10.3389/fneur.2024.1295429

**Published:** 2024-03-28

**Authors:** Jingjing Qiu, JiaZi Xu, Yingyue Cai, Minghong Li, Yingsin Peng, Yunxiang Xu, Guizhen Chen

**Affiliations:** ^1^Shenzhen Bao'an Traditional Chinese Medicine Hospital, Guangzhou University of Chinese Medicine, Shenzhen, China; ^2^Clinical Medical College of Acupuncture-Moxibustion and Rehabilitation, Guangzhou University of Chinese Medicine, Guangzhou, China

**Keywords:** catgut embedding in acupoints, postmenopausal osteoporosis, repetitive transcranial magnetic stimulation, protocol, randomized controlled trial

## Abstract

**Background:**

To date, the clinical modulation for bone metabolism based on the neuro-bone mass regulation theory is still not popular. The stimulation of nerve systems to explore novel treatments for Postmenopausal osteoporosis (PMOP) is urgent and significant. Preliminary research results suggested that changes brain function and structure may play a crucial role in bone metabolism with PMOP. Thus, we set up a clinical trial to investigate the effect of the combination of repetitive transcranial magnetic stimulation (rTMS) and catgut embedding in acupoints (CEA) for PMOP and to elucidate the central mechanism of this neural stimulation in regulating bone metabolism.

**Method:**

This trial is a prospective and randomized controlled trial. 96 PMOP participants will be randomized in a 1:1:1 ratio into a CEA group, an rTMS group, or a combined one. Participants will receive CEA, rTMS, or combined therapy for 3 months with 8 weeks of follow-up. The primary outcomes will be the changes in Bone Mineral Density scores, total efficiency of Chinese Medicine Symptoms before and after treatment. Secondary outcomes include the McGill Pain Questionnaire Short-Form, Osteoporosis Symptom Score, Mini-Mental State Examination, and Beck Depression Inventory-II. The leptin, leptin receptor, and norepinephrine levels of peripheral blood must be measured before and after treatment. Adverse events that occur during the trial will be recorded.

**Discussion:**

CEA achieves brain-bone mass regulation through the bottom-up way of peripheral-central while rTMS achieves it through the top-down stimulation of central-peripheral. CEA combined with rTMS can stimulate the peripheral-central at the same time and promote peripheral bone mass formation. The combination of CEA and rTMS may play a coordinating, synergistic, and side-effect-reducing role, which is of great clinical significance in exploring better treatment options for PMOP.

**Clinical trial registration**: https://www.chictr.org.cn/, identifier ChiCTR2300073863.

## 1 Introduction

Postmenopausal osteoporosis (PMOP) is a systemic metabolic disorder characterized by reduced bone mass, degenerative alterations in bone tissue microstructure, and an elevated risk of bone fragility and fractures. This condition occurs in women after menopause due to ovarian dysfunction and decreased estrogen levels (1). This disease is characterized by back pain, kyphosis, height loss, spinal deformities, and pathological fractures. Osteoporotic fractures significantly contribute to increased morbidity and mortality in postmenopausal women (2). Women have a higher incidence of fractures than men, with a fracture rate twice that of men starting at age 50. Low bone mass is a significant risk factor for fractures in postmenopausal women (3). PMOP is currently a public health problem faced by the world, which not only significantly impacts the physical health and quality of life of patients but also brings a significant economic burden to the healthcare system (4). The direct medical costs of OP in the United States were estimated to be between 13.7 and 20.3 billion dollars in 2005. The price will reach 25.3 billion dollars by 2025 (5, 6).

The clinical treatment methods for PMOP mainly include medication and non-medication (7–9). Bisphosphonates are still the first-line medicine for OP, but potential long-term adverse reactions must be considered (10). A survey found that 19% of postmenopausal women diagnosed with OP did not receive anti-OP drug treatment, and 52% of patients refused the physician’s recommendation for drug treatment, mainly due to concerns about the potential side effects (11). Possible adverse reactions and patient resistance severely limit the clinical prevention and treatment of PMOP. Catgut embedding in acupoints (CEA) combines traditional acupuncture with modern medical theory. Guided by the idea of ‘deep and long-lasting insertion to treat chronic and stubborn diseases’, absorbable surgical sutures were creatively embedded deeply into acupoints during CEA to achieve sustained stimulation. Many clinical practices and studies have confirmed that CEA for PMOP has the advantages of good efficacy, safety, and reliability (12). The CEA has apparent advantages in overcoming and solving expensive medical costs, drug toxicity, and side effects, shortening traditional acupuncture treatment time, and improving patient compliance, which is worth further research (13). However, high-quality evidence from large-scale multicenter clinical randomized trials is still needed to support the treatment outcomes.

The nervous system also plays a regulatory role in bone remodeling and maintenance of bone mass. Scholars have found many neurotransmitters and hormones related to bone remodeling in the brain or brain-related organs and proposed mechanisms for crosstalk of the brain and bone (14–16). The theory of brain-bone mass regulation is of great significance for studying bone metabolism mechanisms. The brain regulates bone mass through neural-bone formation, neural-endocrine, and neuropeptide-bone regulation networks (17). Based on the methods of the functional magnetic resonance imaging (fMRI) technique, we speculated that the brain function and structure may play a crucial role in bone metabolism with PMOP. The CEA may exert its therapeutic effect on PMOP through the functional connection of the hypothalamic nuclei with the frontal cortex (18). CEA is a peripheral stimulation that regulates bone metabolism through the bottom-up, peripheral-central-peripheral pathway.

Repetitive transcranial magnetic stimulation (rTMS) generates a robust and rapidly changing magnetic field that induces current in the target area under the coil, thus activating cortical and subcortical neurons. It regulates neuronal excitability or inhibition, evaluates the corticospinal tract, and enhances brain plasticity (19, 20). This technique has been applied to evaluate the motor system, explore brain function, and investigate the pathophysiology of psychiatric disorders (21, 22). rTMS is currently an essential neuromodulation technique that can be used directly by stimulating the central targets in order to treat neurological diseases and peripheral systemic conditions (23, 24). Based on the neuro-bone mass regulation theory, we propose a central stimulation intervention to regulate bone metabolism by using the neuromodulatory effect of rTMS. We hypothesize that the combination of CEA and rTMS may have a coordinated, synergistic, and reaction-reduced effect, which has significant clinical value in exploring better treatment options for PMOP. The prevention and treatment of PMOP should not rely solely on peripheral interventions but also focus on central involvement. It is essential to integrate central and peripheral regulation for bone remodeling with the participation of the central and peripheral nervous systems.

CEA achieves brain bone mass regulation through the bottom-up peripheral-central way, while rTMS achieves it through the top-down stimulation of the central-peripheral (25). The combination of CEA and rTMS may achieve a bidirectional central-peripheral intervention on bone metabolism (26). Consequently, the study aims to explore the clinical effectiveness and safety of rTMS combined with CEA in treating PMOP from the perspective of central-peripheral regulation. We would like to preliminarily investigate the intrinsic correlation between CEA combined with rTMS, brain bone mass regulation, and clinical signs and elucidate the central mechanism of CEA combined with rTMS in regulating bone metabolism.

## 2 Methods

The study protocol is designed and performed according to the principles of the Declaration of Helsinki and in line with the guidelines of the clinical trial committee for PMOP. The Consolidated Standards of Reporting Trials (CONSORT) statement^1^ has been used to develop the study methodology. This study’s Clinical Trial Registration Number is (ChiCTR2300073863) and has been registered at http://www.chictr.org.cn. The protocol of this study was approved by the Ethics Committee of Bao’an Hospital of Traditional Chinese Medicine, Shenzhen (KY-2023-001-30).

### 2.1 Design and study setting

A total of 96 participants meeting the diagnostic criteria for PMOP according to the guidelines of the clinical trial committee will be recruited at Bao’an Hospital of Traditional Chinese Medicine. Subjects will be informed of every study detail and sign an informed consent form.

**Figure 1:**
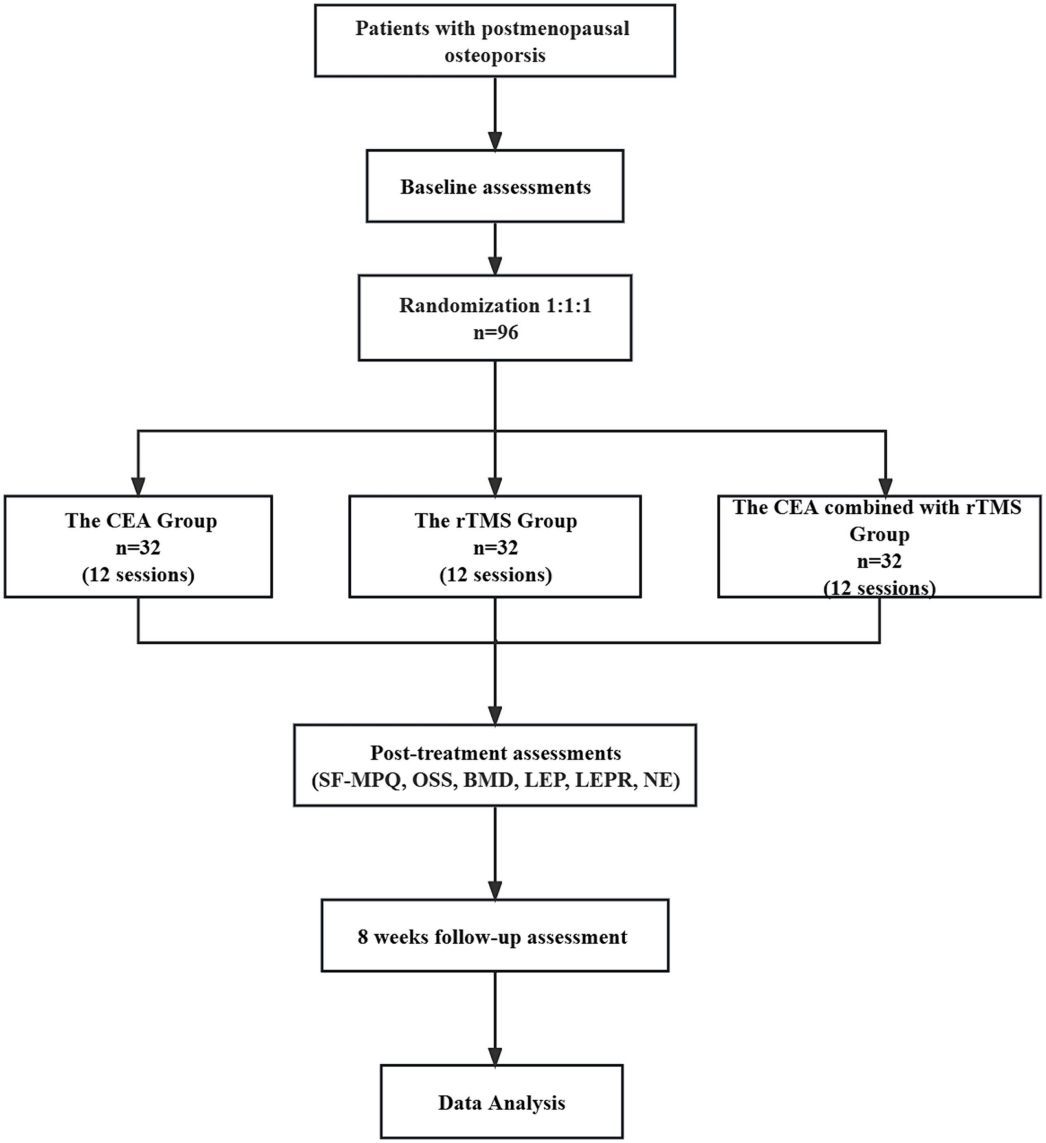
Flow chart.

This randomized, controlled clinical trial comprises three parallel groups. It compares the effectiveness of the individualized CEA group, CEA combined with the rTMS group and rTMS groups (Figure 1).

### 2.2 Participants recruitment

Three ways of recruiting participants will be used: firstly, we intend to recruit the participants from the clinics of Shenzhen Bao’an Hospital of Traditional Chinese Medicine. A workshop will be convened to formulate how to collect participants. In addition, posters will be placed outside the clinic to attract potential candidates. Secondly, Another is recruiting through radio, television, newspapers, etc. In these advertisements, we will introduce the target population briefly and offer free screening and treatment to suitable participants. Thirdly, we will conduct educational talks and offer clinic services to postmenopausal women with OP. By disseminating basic knowledge, we may attract appropriate subjects.

### 2.3 Inclusion criteria

1. Patients who meet the Chinese and Western diagnostic criteria for PMOP. Western medical diagnostic criteria: Referring to the Clinical Practice Manual-Osteoporosis and Bone Mineral Disorders and the Primary Osteoporosis Primary Care Guidelines (2019) from the Chinese Medical Association, as well as the one formulated by American College of Clinical Endocrinologists (AACE) associated with the American College of Endocrinology (ACE) named as the 2020 AACE/ ACE Clinical Practice Guidelines: Diagnosis and Treatment of Postmenopausal Osteoporosis and the World Health Organization (WHO) diagnostic criteria for osteoporosis based on Dual-emission X-ray Absorptiometry (DXA) measurement. The diagnostic criteria for TCM identification are mainly formulated regarding the Guidelines for the Diagnosis and Treatment of postmenopausal osteoporosis (bone impotence) in Chinese Medicine (2019 edition). The Chinese medicine identification criteria is adopted from the Guidelines for Clinical Research on New Chinese Medicines on Kidney Deficiency formulated by the Ministry of Health of China in 2002.
2. Patients with right-handedness; junior secondary school education or above.
3. Age 45–70 years old female with natural menopause for over 2 years.
4. Those who have good compliance and understanding voluntarily join the project and agree to sign the informed consent form.

### 2.4 Exclusion criteria

1. Patients with diseases that do not fall into the category of PMOP or even primary OP, such as other conditions affecting bone metabolism: endocrine disorders (gonadal, adrenal, parathyroid, and thyroid diseases, etc.), immune diseases such as rheumatoid arthritis, gastrointestinal and renal diseases affecting the absorption and regulation of calcium and vitamin D, malignant conditions such as multiple myeloma, long-term use of glucocorticoids or other drugs affecting bone metabolism, various congenital and acquired abnormalities of bone metabolism, etc.;
2. Patients with malignant neoplasms, primary severe diseases of the cardiovascular, cerebrovascular, hepatic, renal, and hematopoietic systems, or either severe skin diseases or psychiatric disorders who are unable to cooperate or adhere to the completion of treatment;
3. Patients who have accepted anti-OP medication (excluding primary therapy) within the previous 6 months or who have been on the long-term and sustained use of drugs that may affect bone calcium metabolism;
4. Patients who have used psychiatric drugs such as antidepressants or anti-anxiety drugs in the past 6 months;
5. Those with structural abnormalities of the brain, such as occupying intracranial lesions, brain malformations, severe strokes (affecting brain structure), etc.;
6. Those with treatment contraindications such as allergy to CEA;
7. Those with contraindications to rTMS treatment: e.g., with aneurysm clips, implanted neurostimulators, pacemakers, automatic defibrillators, cochlear implants (electrodes) or visual foreign bodies, head-mounted with metal materials, etc.;
8. Those with a history of alcohol, drugs, or other kind of substance abuse or dependence within 1 year;
9. Those with creatinine clearance <35 mL/min;
10. Those who are unable to adhere to the treatment.

### 2.5 Drop-out and exclusion criteria

For those who did not complete the trial within 12 weeks±5 days, categorization will be made according to the reason for not completing the trial.

#### 2.5.1 Drop-out

Including patient self-withdrawal due to poor efficacy, intolerance, refusal to account, loss to follow-up, and doctor dissuasion due to poor adherence, comorbidities, specialties, adverse reactions, etc.

#### 2.5.2 Exclusion criteria

a Those who do not meet the inclusion criteria and are mistakenly included;

b Subjects with poor adherence, not following the designed treatment regimen strictly, or whose something would affect the observed results of the study during the trial;

c Subjects with incomplete information on various materials.

#### 2.5.3 Confounding factors

The protocol prohibits a combination of anti-OP drug.

#### 2.5.4 Discontinuation criteria

a Subjects who have experienced a severe adverse reaction or event that prevents them from continuing to be under treatment;

b Subjects who develop severe comorbidity or other systemic serious medical conditions during the trial and are unable to persist;

c Subjects who have poor compliance and do not cooperate with the relevant treatment despite repeated explanations;

d Subjects who request to withdraw from the study on their own;

e Subjects who take other drugs privately during the treatment period, which may interfere with the study results;

f The patient lost during the follow-up period for various reasons.

### 2.6 Randomization and allocation concealment

First, we intend to generate a random number table from 1 to 120 in Excel and made random number cards. Secondly, we paln to mark the random numbers and groups on the cards following the correspondence principle, putting them in opaque envelopes and disrupting the order of the envelopes. Then, all envelopes are to be placed into a box. Outpatients who met the inclusion criteria in the Rehabilitation Department of Shenzhen Bao’an Hospital of Traditional Chinese Medicine can draw one envelope from the box in the order in which they are treated. Individuals receiving an envelope with a number less than or equal to 96 will be eligible to participate in our study. We intend to use Excel to generate a table of random numbers from 1 to 96. Subjects from 1 to 32 are assigned to follow Catgut embedding in acupoints at the CEA group. Subjects from 33 to 65 are designated for the combined treatment group (rTMS combined with the CEA group), while the remaining 32 are allocated to the rTMS group. Patients are randomly divided into three groups, each comprising 32 individuals. Each group is scheduled to receive the corresponding treatment measures.

### 2.7 Blinding

A professional acupuncturist will independently operate the participants in the CEA group, while an independent professional physician is tasked with treating the rTMS group. The patients in the combined treatment group are to be treated by two trained physicians who collaborate to complete the two intervention plans. The treatment therapists of each group could not obtain specific treatment modes for the other two groups. Upon completing the treatments for the three groups, they are to inform the evaluator of the group code corresponding to the participant. The evaluator is to conduct scale testing on the participants and collect and record data in the case report forms, without access to information about the patients’ group allocations. The researchers responsible for statistical calculations are to remain blinded to the group allocations, having access only to the group codes.

### 2.8 Sample size calculation

This randomized controlled study evaluates the effects of three methods on postmenopausal patients with OP. Bone mineral density (BMD), McGill Pain Questionnaire Short-Form (SF-MPQ), Osteoporosis Symptom Score (OSS), Mini-Mental State Examination (MMSE), and Beck Depression Inventory-II (BDI-II), serum leptin, leptin receptor, norepinephrine level will be used as evaluation indicators. According to the preliminary test data, it was calculated that after treatment, the SF-MPQ score (PRI total score) in the CEA group decreased by 17.23 ± 2.84, that in the rTMS group decreased by 16.41 ± 1.77, and that in the CEA combined with rTMS group decreased by 17.40 ± 2.17. The two-sided test value α = 0.05 was used. Test power 1-β = 0.95. Using G*Power3.1.9.7 software and single factor analysis of variance in the F test (27, 28), the required sample size of n = 87 cases was calculated, and the shedding rate was set at 10%. The total sample size was 96 cases, and the required sample size for each group was 32 cases.

### 2.9 Interventions

Treatment strategies will be developed by consensus with experienced acupuncture practitioners and a neurologist. The trial is to include three groups: the CEA group, the combined group, and the rTMS group. The basic supplement regimen is as follows: 1000-1200 mg of calcium per day is recommended, which can be achieved through dietary intake or supplements. It is important to note that dietary calcium intake is recommended, but if dietary intake is insufficient, supplements can be used to compensate. Vitamin D intake of 800–1,000 international units (IU) per day is also recommended (29).

#### 2.9.1 Study schedule

Baseline information on age, menopause age, weight, education, vital signs, and duration of illness will be collected and recorded (Table 1). Then, the primary outcomes will be the changes in BMD scores and total efficiency of Chinese Medicine Symptoms before and after treatment. Secondary outcomes will include the SF-MPQ, OSS, MMSE, and BDI-II every 4 weeks for 12 weeks of treatment and 8 weeks follow-up period. Adverse events that occur during the trial will be recorded. The leptin, leptin receptor, and norepinephrine levels of peripheral blood must be measured before and after treatment.

**Table 1:** Trial flow and schedule: enrollment, interventions, and assessments.

Research stage			Duration of treatment (weeks)				Follow-up period (weeks)	
Inclusion/exclusion criteria			0	4	8	12	4	8
Sign the informed consent form			√					
		General information	√	√	√	√		
		Previous medical history	√					
		Clinical symptoms and physical signs	√	√	√	√		
Physical examination		Tongue manifestation, pulse, manifestation, weight	√			√		
		Vital signs: T, BP, P, R.	√			√		
Security check		Electrocardiogram	√			√		
		Liver function	√			√		
		Renal function	√			√		
		Blood lipids	√			√		
Intervention		CEA						
		rTMS						
		CEA combined with rTMS						
Efficacy indicators	Primary outcome	①BMD	√			√			②Total efficiency of Chinese Medicine Symptoms	√			√		
		③SF-MPQ	√	√	√	√	√	√
		④OSS	√	√	√	√	√	√
	Secondary outcome	⑤Leptin	√			√		
		⑥Norepinephrine	√			√		
		⑦Leptin receptor	√			√		
		⑧MMSE	√	√	√	√	√	√
		⑨BDI-II	√	√	√	√	√	√
Blood sample collection			√			√		
Randomization			√					
General assessment	Clinical efficacy					√		
	Compliance assessment		√	√	√	√		
	Adverse event		√	√	√	√		
	Safety evaluation		√			√		

T, Temperature; BP, Blood Pressure; P, Pulse; R, Respiration; CEA, Catgut Embedding in Acupoints; rTMS, repetitive Transcranial Magnetic Stimulation; BMD, Bone Mineral Density; SF-MPQ, McGill Pain Questionnaire Short-Form; OSS, Osteoporosis Symptom Score; MMSE, Mini-Mental State Examination; BDI-II, Beck Depression Inventory-II.

#### 2.9.2 The CEA group

Our treatment method is based on previous literature reports (30).

The treatment group will undergo 12 weekly sessions of CEA for 3 months. The primary acupoints will be BL23, SP6, and RN4, with additional acupoints like BLI8, GB39, BL11, BL20, and ST36 added, depending on whether the case is a liver-kidney deficiency or kidney yang deficiency. During each treatment, RN4 is essential, while the other acupoints can be alternated between the left and right sides. The acupoint location method will follow the State Bureau of Technology Supervision standards. In addition, the operating procedure will be based on the National Standards of GB/T 21709.10–2008 manipulations of acupuncture and moxibustion, Part 10 Acupoint catgut embedding. Detailed information about the intervention group and acupoint location can be found in Tables 2, 3 of the STRICT checklist for reporting interventions in clinical acupuncture trials (Tables 2, 3).

**Table 2:** Acupoints selection.

Primary point	Additional point	
	Deficiency of liver and kidney	Deficiency of kidney yin
Shenshu (BL23) Sanyinjiao (SP6) Guanyuan (RN4)	Dazhu (BL11) Ganshu (BL18)	Xuanzhong (GB39) Zusanli (ST36) Pishu (BL20)

**Table 3:** Acupoints location.

Point	Location
BL23	1.5 cun lateral to the depression below the spinous process of the second lumbar vertebra
SP6	Posterior to the mesial border of the tibia and 3 cun above the tip of the medial malleolus
RN4	On the anterior midline, 3 cun below the umbilicus
BL18	1.5 cun lateral to the depression below the spinous process of the 9th thoracic vertebra
BL11	1.5 cun lateral to the lower border of the spinous process of the first thoracic vertebra
GB39	3 cun above the tip of the external malleolus, on the anterior border of the fibula
ST36	3 cun directly below Dubi (the lateral depression of the knee-joint) and one middle finger-breadth lateral to the anterior border of the tibia
BL20	1.5 cun lateral to the depression below the spinous process of the 11th thoracic vertebra

Cun=an acupuncture measurement unit; 1 cun corresponds to the study subject’s thumb width.

##### 2.9.2.1 CEA group materials

1. Disposable sterile burying kit: provided by the Shenzhen Bao’an Hospital supply room of Traditional Chinese Medicine.
2. Injection syringe: disposable sterile injection needle seven #(0.7*30 TWLB), Wuhan Wangguan Medical Equipment Co., Ltd.: State Food and Drug Administration Machinery (Quasi) No. 3151148, 2014.
3. Absorbable surgical suture, multi-strand braided structured polyglycolic acid PGA suture, specification: 3–0 (20metric) 1.5 cm/20 segments, [Zhejiang Kandlai Medical Devices Co. No. 3151299].
4. Andover 0.5% PVP-I Disinfectant Solution, Shenzhen Andover Disinfection High-Tech Co: Guangdong Health Disinfection Certificate (2016) No. 9087.
5. Medical Sterilization Swabs, Shun Kangzheng, Model KZ3-12, Foshan Shunde Kangzheng Sanitary Material Co. Company: Guangdong Food and Drug Administration Machinery Production License 20,010,167.
6. Aseptic Dressing, Product No.: DF-needle eye, Zhejiang Chun’an County Renhe Medical Supplies Industry and Trade Co. Zhejiang Food and Drug Administration Machinery (Permitted) Word 2014 No. 2640695.

##### 2.9.2.2 Operating method (implantation with needles)

1. According to National Standards of P.R (GB/T 21709.10-2008), manipulations of acupuncture and moxibustion—Part 10.
2. As per the National Standards of P.R (GB/T 21709.10-2008), implanting needles for acupuncture and moxibustion involves cutting 3/0 thread into 1.5 cm pieces and soaking them in disinfectant.
3. Patients are to be positioned prone for BL23, BL18, BL11, BL20 point implantation and supine for RN4, SP6, GB39, and ST36 implantation. After routine disinfection of the acupoint and surrounding skin with Anerdian, a self-made embedding needle is used to rapidly stick the needle into the acupoint with the right hand while the left thumb and forefinger tighten or pinch the skin around the acupoint. After receiving qi, the core is pushed, and the tubing is withdrawn slowly to complete the thread implantation deep down the acupoint. After checking for exposure of the thread out of the skin and bleeding, the wound is covered with gauze or a band-aid for 1–2 days.
4. The direction, angle, and depth of insertion must be considered to implant the needles into different acupoints properly. Acupoints such as BL23, SP6, RN4, and GB39 require a vertical puncture for approximately 0.8 to 1.0 cun (about 20-25 mm), while ST36 requires a similar punch for about 1.0 to 1.5 cun (about 25–40 mm). BL20 requires a vertical puncture of around 0.5 to 0.8 cun (about 13–20 mm), while acupoints such as BL18 and BL11 are required for proper needle insertion.

#### 2.9.3 The rTMS group

The treatment will be completed by a trained professional therapist using a transcranial magnetic stimulator (YRD CCY-II from Wuhan Eredo). The motor threshold (MT) and dorsolateral frontal lobe (DLPFC) stimulation site are to be determined during the first treatment. The room temperature will be maintained at 16°C–23°C, and the patient will be lying flat on their side on the treatment bed. The motor-evoked potentials (MEPs) are to be recorded in the contralateral hand’s interosseous muscle through the magnetic stimulator’s electromyographic amplifier, with the center of the “8” coil placed on the right temporal cortex of the subject. The stimulation site and the stimulation volume are adjusted until at least 5 out of 10 stimulations evoke MEPs with an amplitude more significant than 50 μV, at which point the magnetic flux is MT, and move 4 cm forward horizontally at the site where the MEPs are elicited, which is the DLPFC. We plan to utilize a positioning cap and a transcranial magnetic stimulator (YRD CCY-II from Wuhan Iredell) to accurately identify and target the specific location during the procedure (31). We intend to choose the right DLPFC point as the stimulation site. The stimulation intensity will be set at 80% of the resting movement threshold, with a frequency of 1 Hz, lasting 30s with an interval of 4 s, and 10 series to be performed continuously on the patient (32, 33). The treatment plan will consist of one session per day, 5 days a week, for 4 weeks (34, 35). Starting from the fifth week, the treatment plan changes to twice a week for eight consecutive weeks (36). In total, this treatment plan will last for 12 weeks and include 36 sessions.

#### 2.9.4 The CEA combined with rTMS group

Subjects in this group will receive both the CEA and the rTMS treatment according to the respective regimens of the two therapies.

#### 2.9.5 Adverse events

##### 2.9.5.1 Mild reactions

1. Local aseptic inflammatory reactions, such as redness, swelling, heat, and pain, often occur within 1–5 days.
2. Systemic reactions may include a rise in body temperature within 4–24 h after treatment and an increase in blood count, which usually returns to normal in 3–5 days.

Most of the mild reactions are physiological after the treatment, do not need to be treated, and can be relieved on their own within a short period.

##### 2.9.5.2 Severe reactions

1. Hot compresses can alleviate pain in the needling points.
2. Local redness, swelling, pain, fever, and other inflammatory manifestations caused by secondary infection can be resolved within 3–4 days.
3. Nerve injury can occur due to incorrect operation, over-stimulation, or carelessness but can be prevented carefully.
4. Bleeding may happen because of puncturing blood vessels or excessive stimulation. Compression bandages can be used to stop bleeding.
5. Local itching, redness, swelling, fever, etc., may be symptoms of an allergy to threads. Anti-allergic treatment can be given in such cases.

Appropriate actions are taken to address more severe reactions in accordance with the grading and management of adverse reactions in CEA (37). Effective measures include discontinuation of the trial or ethically appropriate additional mechanisms.

### 2.10 Outcome measurement: primary outcomes

#### 2.10.1 BMD test

The patient’s BMD of L2-L4 in the lumbar spine and BMD of the left femoral neck will be measured by DXA once before and after the treatment.

#### 2.10.2 Total efficiency of Chinese medicine symptoms

According to the “Guidelines for Clinical Research on New Chinese Medicine,” the efficacy assessment criteria for osteoporosis are as follows:

Significant efficacy: bone density examination shows an increase in bone density.

Effective: significant relief of pain, and bone density examination shows no decrease in bone density.

Ineffective: no improvement in all aspects compared to before treatment.

The efficacy index is calculated by integrating the scores before and after treatment, where the efficacy index = (score after treatment-score before treatment) / score before treatment × 100%.

The overall effective rate = (significant efficacy + effective) /*n* × 100%.

### 2.11 Outcome measurement: secondary outcomes

#### 2.11.1 Clinical manifestation

SF-MPQ and OSS will be measured every four weeks for 5 months. All evaluation indicators will be assessed at six time points: baseline, mid-treatment (4 and 8 weeks of treatment), post-treatment (12 weeks), and 1 month and 2 months after the treatments.

##### 2.11.1.1 SF-MPQ

The first part of the SF-MPQ consists of 15 pain descriptors, of which 11 are items about self-sense and four are about uncomfortable feelings. Each item has three levels, from 0 (painless) to 3 (severe) (Table 4).

**Table 4:** Short-form McGill Pain Questionnaire Part I.

	Pain Rating Index	None (0)	Light (1)	Medium (2)	Heavy (3)
Sensory rating	Throbbing pain				
	Piercing				
	Stabbing pain				
	Sharp pain				
	Cramping pain				
	Colic				
	Hot-burning pain				
	Continuous fixation pain				
	Swelling and pain				
	Pain caused by light touch				
	Splitting pain				
Emotional rating	Tiring-exhausting				
	Sickening				
	Fearful				
	Punishing-cruel				

**Figure 2:**
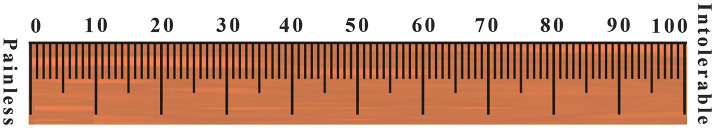
The VAS scale.

The second part is a 10-centimeters-long visual assessment scale (VAS), marked as “painless” at one end and “intolerable pain” at the other end, on which the participants draw the level of pain (Figure 2).

The third part is the present pain level (PPI), generally used to indicate pain intensity on a scale. The Scale has five levels, from 0 (painless) to 5 (intolerable pain). PMOP patients were evaluated before and after treatment, including the current pain and how pain has been felt in the past week (Table 5).

**Table 5:** Present pain intensity.

Pain Level	Description of pain intensity
0	Painless
1	Mild pain (occasionally annoying due to pain)
2	Moderate pain (often annoying but tolerable with restraint)
3	Severe pain (can only tolerate partial pain)
4	Terrible pain (severe pain, often causing moaning)
5	Unbearable pain (feeling the pain too much to commit suicide)

##### 2.11.1.2 OSS

According to the quantitative criteria for grading the significant clinical symptoms of OP in the “Technical Guidelines for Clinical Studies on New Chinese Medicines for Primary Osteoporosis.” The subjects are assessed on a scale according to time points (Table 6).

**Table 6:** Quantitative criteria for clinical main symptom grading of OP.

Symptom	Light (score 1 points)	Medium (score 2 points)	Heavy (score 3 points)
Pain in the lower back***	1 ~ 3 degree	4 ~ 6 degree	7 ~ 10 degree
Soreness and weakness of waist and knees**	Occur after walking over 1 km	Occur after walking 300 ~ 1 km	Occur after walking under 300 m
Leg cramps**	Occur occasionally at night	Occur frequently at night	Occur frequently at day and night
Trudge*	Short-distance walking without discomfort within 100 m	Difficulty in walking short distances (10–100 meters)	Difficulty in walking (unable to exceed 10 m), or standing
Sedentary difficulty*	Less power to hold heavy	The intermediate state	Completely no power to hold heavy

① Pain level can generally be assessed by the VAS pain scale method; ② *** means score x 3; ** means score x 2; * means score x 1; ③ Subjects use diary cards to record symptoms, and the doctor communicates with the subjects after evaluation.

#### 2.11.2 Clinical objective indicators

The secondary outcome measures are (i) Leptin, (ii) Leptin Receptor (iii) Noradrenaline. The leptin, leptin receptor, and norepinephrine levels of peripheral blood must be measured before and after treatment.

Blood samples should be collected on the day of entry and the first day after the end of treatment for the CEA group, the rTMS group, and the CEA combined with rTMS group. Before sampling, all required tubes should be prepared and appropriately labeled with the study protocol number, subject number, study phase, period, and date and time of sampling. The authorized research nurse is responsible for collecting the blood specimens. After collection, the tubes are inverted and mixed. Blood is drawn in the order required by the collection process to prevent cross-contamination between different types of boxes. If a tube is contaminated, it should be replaced immediately with a spare tube and re-labeled. Vital signs are recorded before sampling. Blood is drawn from a venous elbow at 8:00 am in the fasting state. 3 mL of blood is collected in a pre-prepared tube, and after being centrifuged, the serum is collected for testing. 5 mL of blood is contained in a pre-prepared anticoagulation tube (20 μL/mL of peptidase, 20 μL/mL of 10% EDTA-Na2), centrifuged, and plasma collected for testing. Collect 5 mL of blood in a pre-prepared anticoagulation tube (20 μL of peptidase, 20 μL of 10% EDTA-Na2); after being centrifuged, the plasma is collected for testing.

### 2.12 Measures to prevent excessive loss of cases

1. Communicate in detail with patients about the trial process, provide adequate information, clarify their responsibilities and obligations and the importance of the trial, and sign the informed consent form.
2. Simplify the trial process as much as possible to reduce the burden on subjects. Reasonably arrange the time for data collection and treatment of subjects.
3. Actively respond to patients’ feedback during treatment to improve patient compliance.
4. Develop standard operating procedures for data collection and measurement of subjective and objective indicators and strengthen training for investigators in data collection, communication skills, and scoring operations, which can improve patient compliance and reduce the incidence of missing data.
5. Establish a comprehensive case tracking system to ensure that case participation and follow-up can be effectively managed and recorded.

### 2.13 Safety assessment

Current studies about rTMS have shown few side effects. Given that this study is a low-risk intervention, small sample clinical mechanism research trial, there is no need for a dedicated data safety and monitoring committee. To ensure the rights of participants and the smooth running of the trial, we have developed a data safety and monitoring plan based on the specificity of the disease, subject population, interventions, and outcome indicators of the study. After review by the Ethics Committee, safety inspectors are appointed to track, record, report, and deal with adverse events.

Therapists need to follow the CEA process strictly to ensure the concept of sterility and safety. Comply with the graded treatment of adverse reactions to CEA, assess and effectively identify the grade of adverse reactions in patients promptly, reduce the occurrence of adverse reactions and attenuate their harm, and deal with them promptly (37). Strictly exclude contraindications to rTMS treatment, sign an informed consent form, and complete a safety screening assessment. We intend to select the intensity, frequency, and number of rTMS stimulations according to the treatment purpose since the individual differences in resting motor threshold (RMT) determination. It should be strictly limited to safe sequences and avoid sequences that induce seizures and other risks. The assessment and effective management of adverse effects during treatment should be strictly controlled. Although the current rTMS treatment has a low risk, it is still necessary to prevent rTMS-induced seizures. It has been reported that rTMS can induce seizures in both standard and epileptic patients when the stimulation frequency is between 10 and 25 Hz and the stimulation intensity is above the threshold (38). For example, if a patient experiences a seizure, treatment should be stopped immediately, and first aid should be administered. Application of rTMS near the affected ear should be avoided in patients with hearing symptoms (e.g., tinnitus or phantom hearing).

### 2.14 Statistical analysis

The data will be recorded and checked for accuracy, and the data related to baseline and clinical symptoms will be statistically analyzed using SPSS 27.0 software. The *t*-test will be performed for customarily distributed measures that meet the chi-squared test, and the *t′*test will be conducted for standards that meet the normal distribution but do not meet the chi-squared test. Independent sample *t*-tests are used between two groups, and paired sample *t*-tests will be used for pre-treatment and post-treatment comparisons within groups. Non-normally distributed measures are described by “median (interquartile range)” [*M (IQR)*], and non-parametric tests will be performed. *The Mann–Whitney U* test will compare two independent samples, and the *Wilcoxon Z* test will be used for pre-and post-treatment-comparisons. The scale scores at more than three of these time points are compared using repeated measures *ANOVA*. Correlations will be expressed as correlation coefficients (*r*). Correlations between the two measurement variables will be analyzed by Pearson correlation analysis if they conform to a normal distribution and Spearman analysis if they do not obey a normal distribution. Given that our study defines two primary outcomes, a Bonferroni correction will be applied, thus adjusting the significance level for individual outcome analyzes to *α*/2 to control the overall type I error rate, setting *α* = 0.05 (39).

### 2.15 Dissemination

The study findings will be shared at scientific conferences and published in peer-reviewed journals. The study participants will also have the chance to receive the results through phone or email.

### 2.16 Trial status

Currently, the protocol is version 1.0, registered on 24 July 2023. Clinical registrations have been reviewed and approved, and we are now recruiting subjects.

## 3 Discussion

### 3.1 Feasibility analysis of rTMS combined with CEA for PMOP

The “brain-bone axis” is critical for bone metabolism, sensory innervation and endocrine connections between organs (1). The bone is rich in sensory and sympathetic innervation and interacts with the central nervous system (40, 41). The central regulation of bone mass can be divided into the following three main pathways: i) regulation of the sympathetic nervous system through neuronal signaling in the brainstem and hypothalamus; ii) the hypothalamus-pituitary neuroendocrine signaling pathway; and iii) direct action on bone cells through the hypothalamic secretion of neuropeptides (42). There is growing evidence that the nervous system plays an irreplaceable role in bone development and metabolism by directly or indirectly regulating the activity of osteoblasts (OBs) and osteoclastss (OCs) (43). Thus, based on the neuro-bone mass regulation theory, we aim to stimulate the nerve systems to explore novel treatments for PMOP.

A meta-analysis of our pre-published (44) showed that the efficacy of CEA for PMOP is equivalent to that of the drug control group and is safer. Our previous clinical trials have proved that CEA can increase serum estradiol levels and regulate bone metabolism and free radical levels in PMOP (12). Animal studies have demonstrated that CEA can alleviate oxidative stress resulting from estrogen deficiency. It exerted a regulatory effect on aromatic amino acids, specifically phenylalanine and tyrosine, and may influence the synthesis of monoamine neurotransmitters in ovariectomized rats. Notably, this regulatory effect appeared to surpass that of estrogen (45). Acupoint stimulation of bilateral Shenshu (BL 23) can increase pituitary ERα expression, reduce body weight in ovariectomized rats, and benefit the rats (46). In conclusion, Acupoint stimulation in the periphery may improve bone metabolism by modulating the level of central neurotransmitters (12).

Clinical studies have demonstrated that acupuncture with TMS therapy is more effective than monotherapy in improving clinical symptoms (47, 48). Acupuncture activates various brain regions through a bottom-up modulation approach, while TMS generates action potentials in cortical axons that spread to other neurons via synapses, resulting in neuronal activation that spreads excitation to neighboring cortical and subcortical regions (49). The combination of these two treatments creates a pattern of central-peripheral and closed-loop stimulation that is superior for improving neurophysiological function (50, 51). CEA combined with rTMS can stimulate the peripheral-central simultaneously, thus correcting the imbalance of brain bone mass regulation and promoting peripheral bone mass formation (52–54). The combination of CEA and rTMS may play a coordinating, synergistic, and side-effect-reducing role (55), which is of great clinical value in exploring better treatment options for PMOP.

### 3.2 Stimulating location of intervention modalities for PMOP

Prior research has indicated that bone loss is interconnected with alterations in the structure of the brain (56–60). We chose the DLPFC as the stimulating brain area based on the preliminary results of our clinical study of neuroimaging. Our previous clinical study found that the function and structure of the frontal cortex were altered in patients with PMOP. This alteration was significantly correlated with clinical symptoms. CEA may correct the bone metabolic imbalance by promoting restoring frontal cortex function in PMOP patients (18). rTMS is an effective means of stimulating the cerebral cortex for neuromodulation of the periphery (61). Based on the brain-bone mass regulation theory, we tried to use r-TMS to directly stimulate the frontal cortex to see whether peripheral bone metabolism would be altered. The DLPFC plays a vital role in the whole frontal network. It has been demonstrated that the DLPFC is associated with executing functions such as cognitive control, emotion regulation, and autonomic nervous system modulation (62). This region has been linked to autonomic nervous system modulation and endogenous pain inhibitory mechanisms (63). Based on the neuro-bone mass regulation theory, the neural networks in this region associated with the neuromodulation of bone metabolism may be an ideal stimulation target (64, 65). The DLPFC was chosen for stimulation to target effects on neural circuits associated with bone metabolic regulation (66). This option promotes restoring frontal cortex function by stimulating the right DLPFC, thereby correcting bone metabolic imbalances and improving clinical symptoms in PMOP patients.

Additionally, the technical feasibility and safety of rTMS are key factors. The right DLPFC was chosen based on its relative ease of localization and stimulation, thus ensuring the reproducibility of the trial and the accuracy of the results. Based on the brain bone mass regulation theory, this brain region is a compelling target for CEA in treating PMOP. We have reason to believe that by rTMS stimulation of this area and the effect mechanism of CEA, we can achieve bidirectional central and peripheral bone mass regulation.

### 3.3 Analysis of the selection of PMOP-related scales

PMOP is often called the “silent killer” due to its high incidence and elusive nature, making it difficult for patients to detect and make decisions promptly. Therefore, clinical assessment through a reliable and efficient scale is crucial. The SF-MPQ scale is a reliable, objective, and sensitive evaluation method that can score patients’ pain characteristics, sensory features, emotional factors, etc., especially for assessing osteoporotic pain (67, 68). The scale includes emotional assessment items, such as weakness. Research has shown that OP and mental illnesses have similar biological pathways and common risk factors, such as old age, lack of physical activity, weight loss, and cognitive decline (69, 70). The symptom score of OP has a grading quantification feature, which is more detailed and scientific than other scales, and can evaluate OP-related symptoms from multiple dimensions.

Mental illnesses such as depression and anxiety are closely related to PMOP and need to be studied (71). There is a bidirectional regulatory relationship between OP and depression. On the one hand, OP causes physical pain and discomfort, leading to depression (72). On the other hand, depression accelerates the progression of OP (73). Emotional disorders such as depression and anxiety are closely related to bone metabolism. Studies have shown that chronic stress-induced emotional abnormalities cause bone loss through central nervous systems such as GABAergic neural circuits, sympathetic nervous systems, and glutamatergic neurons (74). CEA may also relieve emotional disorders such as anxiety and somatic anxiety by correcting the balance of excitatory and inhibitory amino acid neurotransmitters, which can affect the bone metabolism and play a role in treating PMOP (75). Depression is a risk factor for decreased BMD and can affect bone absorption and reconstruction processes by stimulating the sympathetic nervous system (SNS), leading to bone loss and OP. As an endocrine organ, bone can secrete bone factors that act on the hypothalamus, inducing depressive symptoms (76). The risk of cognitive impairment increases in postmenopausal women with OP (77, 78). Positive intervention can prevent or delay the occurrence of cognitive impairment in high-risk individuals. Consequently, We use the BDI-II and the MMSE to assess the psychological status and cognitive function in PMOP patients (79–82).

### 3.4 An exploration of the mechanisms of rTMS combined with CEA intervention in PMOP

Studies have shown that the brain-bone mass regulation is closely linked to leptin signaling (83). Leptin deficiency or resistance was associated with high BMD in mice (84). Leptin inhibits the increase in bone mass by suppressing brain-derived 5-hydroxytryptamine (5-HT) synthesis and serotonergic neuronal activity. In addition to this, the leptin increases sympathetic activity in the hypothalamus and releases the neurotransmitter norepinephrine (85). Norepinephrine binds to beta2 adrenergic receptors in bone, inhibiting bone-forming cells (OBs) activity (86). The central nervous system acts on adrenergic signaling to increase beta-adrenergic activity, reducing bone mass (87, 88). Epidemiological studies have shown that beta-adrenergic receptor blockers reduce fracture risk and increase BMD (89, 90).

In an aging or estrogen-deficient state, sympathetic excitability increases, and parasympathetic excitability decreases, resulting in an increase in norepinephrine and a decrease in acetylcholine (Ach) content in bone tissue, which stimulates osteoblast production of neuropeptide Y (NPY) by acting on the osteoclast surface receptor β2AR (91). Kajimura et al. (92) found that brain leptin signaling inhibited CREB (cAMP-reactive element binding protein) phosphorylation via sympathetic signaling in OBs, thereby inhibiting osteoblast proliferation while promoting activating transcription factor 4 phosphorylation, increasing Receptor Activator of Nuclear Factor-κB Ligand expression, stimulating osteoblast proliferation and differentiation, and reducing bone shape to promote bone resorption. Thus, leptin can reduce bone mass by regulating sympathetic activity (93). There is a complex interaction between sympathetic nerves, leptin, and norepinephrine, which can interact in several ways to influence bone metabolism.

Studies have shown that intervention with rTMS in DLPFC results in a corresponding change in leptin levels and that TMS may suppress food impulsivity in bulimic patients by modulating leptin levels (94). TMS has been found to modulate the neuroendocrine system, particularly leptin, by enhancing the PFC’s inhibitory capacity, which in turn effectively reduces BMI and impulsivity (94). Electroacupuncture altered the strength of resting functional connectivity in two brain regions, dorsal caudate and precuneus, in obese patients. This change negatively correlated with lower leptin levels and body mass index (95). Studies have shown that CEA can achieve weight loss by modulating leptin levels in the hypothalamus and solitary tract nucleus and the MAPK pathway in the prefrontal cortex of obese mice (96). Therefore, we speculate that stimulation of the DLPFC by low-frequency rTMS combined with CEA therapy reduces leptin secretion, inhibits sympathetic activity, and reduces norepinephrine secretion, thereby regulating bone metabolism.

## Ethics statement

This study was approved by the Medical Ethics Committee of Shenzhen Bao’an Hospital of Traditional Chinese Medicine (KY-2023-001-30). The trial was registered at https://www.chictr.org.cn/ (ChiCTR2300073863) on 24 July 2023.

## Author contributions

JQ: Conceptualization, Data curation, Formal analysis, Methodology, Resources, Software, Validation, Writing – original draft, Writing – review & editing. JX: Data curation, Formal analysis, Resources, Software, Visualization, Writing – original draft. YC: Data curation, Formal analysis, Methodology, Writing – original draft. ML: Conceptualization, Data curation, Formal analysis, Visualization, Writing – original draft. YP: Conceptualization, Data curation, Writing – original draft. YX: Conceptualization, Investigation, Methodology, Project administration, Visualization, Writing – original draft, Writing – review & editing, Funding acquisition, Supervision. GC: Conceptualization, Funding acquisition, Investigation, Methodology, Project administration, Resources, Supervision, Validation, Writing – review & editing, Writing – original draft.

## Footnotes

^1^ https://www.consort-spirit.org/
